# Regulation of TGFβ in the immune system: An emerging role for integrins and dendritic cells

**DOI:** 10.1016/j.imbio.2012.06.009

**Published:** 2012-12

**Authors:** John J. Worthington, Thomas M. Fenton, Beata I. Czajkowska, Joanna E. Klementowicz, Mark A. Travis

**Affiliations:** a Manchester Immunology Group, Faculty of Life Sciences, AV Hill Building, University of Manchester, Oxford Road, Manchester M13 9PT, UK; b Wellcome Trust Centre for Cell-Matrix Research, Faculty of Life Sciences, AV Hill Building, University of Manchester, Oxford Road, Manchester M13 9PT, UK

**Keywords:** TGFβ, transforming growth factor β, BMP, bone morphogenetic protein, GDF, growth and differentiation factor, TGFβR, TGFβ receptor, LAP, latency-associated peptide, Treg, regulatory T-cell, nTreg, natural regulatory T-cell, iTreg, induced regulatory T-cell, EAE, experimental autoimmune encephalitis, DC, dendritic cell, LC, Langerhans cells, AHR, airway hyper-responsiveness, Adaptive immunity, Dendritic cell, Integrin, T-cell, TGFβ

## Abstract

Regulation of an immune response requires complex crosstalk between cells of the innate and adaptive immune systems, *via* both cell–cell contact and secretion of cytokines. An important cytokine with a broad regulatory role in the immune system is transforming growth factor-β (TGF-β). TGF-β is produced by and has effects on many different cells of the immune system, and plays fundamental roles in the regulation of immune responses during homeostasis, infection and disease. Although many cells can produce TGFβ, it is always produced as an inactive complex that must be activated to bind to the TGFβ receptor complex and promote downstream signalling. Thus, regulation of TGFβ activation is a crucial step in controlling TGFβ function. This review will discuss how TGFβ controls diverse immune responses and how TGFβ function is regulated, with a focus on recent work highlighting a critical role for the integrin αvβ8 expressed by dendritic cells in activating TGFβ.

## TGFβ structure and signalling

**Fig. 1:**
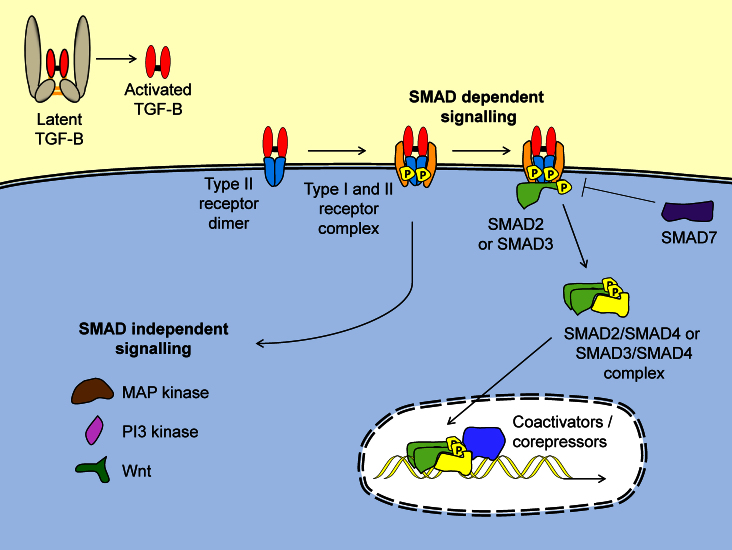
Activated TGFβ signals *via* Smad-dependent and independent pathways. Once the latent complex of TGFβ is activated, active TGFβ binds to TGFβRII, resulting in recruitment of a TGFβRI. Phosphorylation of TGFβRI by TGFβRII allows either SMAD2 or SMAD3 to bind. TGFβRI phosphorylates SMAD2/3, which dissociates from the receptor complex and binds SMAD4. SMAD7 is able to disrupt SMAD2/3 attachment to the receptor complex. SMAD4 in complex with SMAD2/3 translocates to the nucleus where they decorate SMAD-binding elements of gene promoter regions. Constitutive dephosphorylation of SMADs results in their export from the nucleus, ensuring tight regulation of TGFβ signalling. The complex is able to recruit coactivators or corepressors to either up- or down-regulate expression of a wide range of genes, dependent on the cell type. TGFβ can also signal through poorly understood SMAD-independent pathways including MAP kinase, PI3 kinase, Wnt and other pathways.

TGFβ is the prototypical member of the TGFβ family of cytokines, which consists of 33 members and includes the activins, inhibins, bone morphogenetic proteins (BMPs) and growth and differentiation factors (GDFs) (Derynck and Miyazono 2008). There are three isoforms of TGFβ (TGFβ1, 2 and 3), all of which have overlapping but non-redundant functions (Annes et al. 2003). Importantly, all three TGFβ isoforms are produced as inactive complexes, which must be activated to bind to their receptors. Thus, TGFβ genes encode a single product consisting of an N-terminal propeptide called latency associated peptide (LAP) and a C-terminal active cytokine moiety (herein referred to as active TGFβ). After translation, the LAP-active TGFβ product dimerises *via* disulphide bond formation. LAP and active TGFβ are then cleaved from each other in the Golgi by the enzyme furin, but remain non-covalently associated in a conformation that blocks active TGFβ binding to its receptor (Fig. 1) (Annes et al. 2003). Recent structural data show that LAP forms the arms of a ‘straightjacket’ that wrap around active TGFβ to mask the receptor binding sites and keep the complex in an inactive form (Shi et al. 2011). Thus, TGFβ can only signal *via* the TGFβ receptor and trigger biological effects once the complex is activated.

The TGFβ receptor complex consists of two receptor subunits, TGFβ receptor (TGFβR) I and II. Initial engagement of active TGFβ with TGFβRII causes a conformational change in the receptor which facilitates dimerisation with TGFβ RI (Fig. 1). A dimer of TGFβRII and TGFβRI forms a tetrameric complex bound to the active TGFβ dimer, to initiate downstream signalling pathways (Kang et al. 2009).

Classically, TGFβ receptor signalling occurs by activating the Smad-dependent intracellular signalling pathway. Both TGFβRI and II are serine-threonine kinases, and upon formation of the active TGFβ–TGFβR complex, the cytoplasmic domain of TGFβRII phosphorylates the cytoplasmic domain of TGFβRI, which recruits either Smad2 or Smad3 to TGFβRI. Smad2/3 is then phosphorylated by TGFβRI, and a homodimer binds to Smad4 before shuttling to the nucleus where it acts as a transcription factor to regulate gene transcription (Fig. 1) (Shi and Massagué 2003). The interaction of Smad4 with Smad2/3 can be blocked by the inhibitory Smad, Smad7, which can act as an important negative regulator of TGFβ signalling (Monteleone et al. 2008). However, TGFβ can also propagate signals *via* a number of Smad-independent routes including the MAP kinase, PI3-Kinase and Wnt pathways (Guo and Wang 2009). Thus, as TGFβ can regulate numerous intracellular signalling pathways and many cells express TGFβRs, this pleiotropic cytokine needs to be carefully regulated. As will be discussed, a key regulatory step in the TGFβ signalling pathway is at the level of cytokine activation.

## Regulation of immunity by TGFβ

Although TGFβ can have diverse effects on multiple cell types, the cytokine plays a non-redundant, crucial role in regulating immunity. TGFβ is produced by, and has diverse functional effects on, many cells of the immune system. The most highly expressed isoform of TGFβ in the immune system is TGFβ1, and mice lacking this isoform die early in life from multi-organ inflammation (Shull et al. 1992; Kulkarni et al. 1993). Thus, TGFβ has classically been proposed to have important anti-inflammatory effects in the immune system. However, it is clear that TGFβ can have both pro- and anti-inflammatory effects depending on the context in which it is acting. Although it should be noted that virtually all cells of the immune system can produce and respond to TGFβ to some extent (see Li et al. 2006a for detailed review), below we highlight the important roles of TGFβ in regulating T-cells and dendritic cells, the key players in driving the adaptive immune response.

## TGFβ regulation of T-cells

### CD4+ T-cells

A major focus has been placed on the role of TGFβ in regulation of CD4+ T-cell responses. Multi-organ inflammation observed in TGFβ1−/− mice is completely rescued if mice are crossed to a MHCII knockout background, highlighting a crucial role for TGFβ in regulating pathological CD4+ T-cell responses (Letterio et al. 1996). Additionally, early *in vitro* evidence suggested that TGFβ can directly downregulate both Th1 and Th2 cell differentiation by suppressing T-bet and GATA-3 expression respectively (Li et al. 2006a). More recently, generation of mice lacking TGFβRII specifically on T-cells has re-inforced the importance of TGFβ in regulating T-cell responses *in vivo*, as mice develop multi-organ inflammation similar to that seen in TGFβ1−/− mice (Li et al. 2006b; Marie et al. 2006). Disease development correlated with an enhanced Th1 response; however, in the absence of the Th1 master transcription factor T-bet, mice lacking TGFβRII on T-cells developed multi-organ inflammation associated with an enhanced Th2 response (Li et al. 2006b). Together, the data support a key role for TGFβ in attenuating both Th1 and Th2 CD4+ T-cell activation to maintain immune homeostasis.

In addition to the direct effects on effector Th1 and Th2 responses, TGFβ can promote immunosuppression *via* direct induction of regulatory T-cells (Tregs), a key CD4+ T-cell subset involved in the suppression of self-harmful immune responses. Tregs are either produced in the thymus during T-cell development (natural or nTregs) or induced from naive CD4+ T-cells in the periphery (so-called induced or iTregs) and are marked by expression of the transcription factor Foxp3 (Sakaguchi and Powrie 2007). Although earlier evidence suggested that TGFβ played no role in the development of nTreg in the thymus (Marie et al. 2005, 2006; Li et al. 2006b), it now appears that TGFβ is involved in the early differentiation of T-cells to nTregs in the thymus. Thus, neonatal mice lacking TGFβRI on T-cells show reduced levels of thymic Foxp3+ Tregs, but these are expanded by enhanced IL-2 production in the first week of life (Liu et al. 2008).

However, it is clear that TGFβ plays a fundamental role in the induction of iTregs in the periphery. In combination with IL-2 *in vitro*, TGFβ directly promotes expression of Foxp3 in CD4+ T-cells, converting them to a regulatory phenotype (Chen et al. 2003). Both TGFβ1−/− mice and mice lacking TGFβRII on T-cells show reduced Foxp3+ Treg numbers in the periphery (Marie et al. 2005, 2006; Li et al. 2006b), suggesting a key role for TGFβ in induction/maintenance of Tregs *in vivo*. Induction of iTregs is particularly prevalent in the intestine, where they are required to prevent reactions against commensal bacteria and innocuous food antigens. In addition to induction and maintenance of Foxp3 expression, TGFβ has also been shown to be important in the functional ability of Tregs to suppress immune responses (Marie et al. 2005; Fahlén et al. 2005).

In contrast to the negative regulatory role in CD4+ T-cell responses, TGFβ has been shown to be a key cytokine in the differentiation of pro-inflammatory Th17 cells in mice and humans (Littman and Rudensky 2010). In combination with the inflammatory cytokines IL-6, IL-1β IL-21 and/or IL-23, TGFβ promotes expression of the master transcription factor of Th17 cells, RORγt (Korn et al. 2007). Although recent work has suggested that Th17 cells can differentiate into potent effector cells in the absence of TGFβ (Ghoreschi et al. 2010), many studies have demonstrated an important role for TGFβ in development of Th17 cells *in vivo*. For example, blockade of TGFβ signalling in T-cells (*via* expression of a dominant-negative TGFβRII) or local administration of anti-TGFβ blocking antibodies prevents the development of Th17 cells and protects mice from disease during experimental autoimmune encephalitis (EAE) (Veldhoen et al. 2006). Similarly, mice lacking TGFβ production in T-cells show reduced differentiation of Th17 cells and protection from EAE (Li et al. 2007). Indeed, it appears that Th17 cells themselves are an important source of TGFβ that acts in an autocrine manner to maintain Th17 cells *in vivo* (Gutcher et al. 2011).

In addition to promoting Th17 cell responses recent data suggest that, in combination with IL-4, TGFβ can drive differentiation of a novel CD4+ effector T-cell subset called Th9 cells, which are characterised by expression of IL-9 (Dardalhon et al. 2008; Veldhoen et al. 2008). In the presence of IL-4, TGFβ induces expression of the transcription factor PU.1, which can directly drive expression of IL-9 (Chang et al. 2010). Th9 cells have effector properties and are involved in responses to helminth infection and induction of tissue inflammation (Dardalhon et al. 2008; Veldhoen et al. 2008). Thus, it is clear that TGFβ can both inhibit and promote differentiation of effector CD4+ Th cell subsets.

Interestingly, the levels of active TGFβ present during an immune response may be important in determining whether TGFβ promotes or inhibits T-cell responses. At low concentrations, TGFβ synergises with pro-inflammatory cytokines to promote Th17 differentiation by inducing RORγt expression (Zhou et al. 2008). However, at higher concentrations TGFβ promotes Foxp3 induction and iTreg formation (Zhou et al. 2008). Thus, although the significance *in vivo* remains to be determined, these data suggest that alterations in local active TGFβ concentrations may have profound effects on whether CD4+ T-cells are pushed towards a suppressive or effector phenotype.

### Other T-cell subsets

In addition to regulating CD4+ T-cell subsets, TGFβ plays important roles in controlling other T-cell compartments. Thus, TGFβ signalling is involved in CD8+ T-cell development in the thymus, and restrains CD8+ T-cell activation in the periphery of mice (Li et al. 2006b; Marie et al. 2006). Also, TGFβ limits the expansion of CD8+ T-cells during *Listeria monocytogenes* infection by inducing apoptosis during clonal expansion (Sanjabi et al. 2009). TGFβ signalling is also important for NKT cell homeostasis and is required for the thymic development of canonical CD1d-restricted NKT cells, sometimes referred to as invariant NKT (iNKT) cells (Li et al. 2006b; Marie et al. 2006; Doisne et al. 2009; Havenar-Daughton et al. 2012). Conversely, TGFβ signalling is required for the suppression of pathogenic effector function in an NK1.1^+^ T-cell subset (Marie et al. 2006). Finally, TGFβ has recently been shown to be critical in the formation of CD8αα+ intraepithelial lymphocytes in the intestine, which are proposed to be important in regulation of mucosal immune responses (Konkel et al. 2011).

Taken together, it is clear that TGFβ plays essential roles in the regulation of T-cell development and function, acting to tune immune responses by promoting or suppressing different subsets during homeostasis and infection.

## TGFβ regulation of dendritic cells

The action of TGFβ on dendritic cells (DCs) can both promote and inhibit DC-mediated immune responses. TGFβ can downregulate the antigen-presenting function and expression of co-stimulatory molecules by DCs *in vitro* (Strobl and Knapp 1999). Mice expressing a dominant negative version of TGFβRII under the control of the CD11c promoter do not show any differences in DCs during homeostasis (Laouar et al. 2005) but are more prone to the development of EAE due to enhanced Th1 and Th17 responses (Laouar et al. 2008). Thus, TGFβ appears to be important in regulating the ability of DCs to control self-reactive T-cells during models of autoimmunity. However, the exact mechanisms by which TGFβ regulate DC-mediated responses *in vivo* require further exploration.

Although TGFβ does not appear to be important in the development and homeostasis of conventional DCs, TGFβ plays an important role in the homeostasis of Langerhans cells (LCs), a unique subset of DCs found in the epidermis of the skin (Kaplan 2010). TGFβ1−/− mice completely lack LCs (Borkowski et al. 1996), suggesting an important role for TGFβ in the development/maintenance of LCs in the skin. Indeed, subsequent work has shown that TGFβ1 production by LCs acting in an autocrine manner is important for LC homeostasis, as expression of a dominant negative TGFβRII in LCs (Kaplan et al. 2007) or deletion of the TGFβRI specifically in LCs (Zahner et al. 2011) results in very low LC numbers in mice. Ablation of TGFβ signalling in total skin DCs (by crossing mice expressing a conditional allele of the TGFβRI receptor to mice expressing CD11c-Cre) confirmed that TGFβ is required to maintain LCs in skin (Kel et al. 2010). The authors showed that TGFβRI deletion in skin DCs was incomplete in neonatal mice, but increased in the first week of life and correlated with a reduction in LC numbers (Kel et al. 2010). Lack of TGFβ signalling in skin DCs also enhanced LC migration and maturation in steady state (Kel et al. 2010). Together, the data suggest that TGFβ production and signalling are crucial for the development, maintenance and function of LCs *in vivo*.

## TGFβ production in the immune system

Many different immune cell types are capable of producing TGFβ (Li et al. 2006a). Studies to investigate the important cellular sources of TGFβ *in vivo* have highlighted an important role for T-cell produced TGFβ. Thus, conditional deletion of TGFβ1 expression in CD4+ and CD8+ T-cells (by crossing mice carrying a conditional floxed allele of TGFβ1 with mice expressing CD4-Cre) resulted in age-related autoimmune disease, characterised by T-cell activation and colitis (Li et al. 2007). This phenotype is similar to that seen in mice expressing a dominant negative TGFβRII in T-cells (Gorelik and Flavell 2000) suggesting that TGFβ acts upon T-cells in an autocrine fashion. However, as the autoimmune phenotype observed is not as severe as that in either TGFβ1−/− mice or mice completely lacking TGFβRII expression in T-cells, this suggests that there are other important cellular sources of TGFβ that act to control T-cells. Indeed, to date, TGFβ knockout in any one single cell type has not recapitulated the severe phenotype observed in TGFβ1−/− mice and mice lacking TGFβRII in T-cells, strongly suggesting that there are multiple important cellular sources of TGFβ production *in vivo*. The autocrine function of TGFβ1 in both T cells (Li et al. 2007; Gutcher et al. 2011) and LC (Kaplan et al. 2007) suggests that TGFβ often acts over short distances on local cells, which may be an important mechanism of spatially restricting the actions of this potent, multi-functional cytokine.

## Activation of TGFβ in the immune system- a key role for integrins

As TGFβ is secreted as an inactive complex, regulation of TGFβ activation is crucial in controlling TGFβ function. Early data showed that the latent complex of TGFβ could be activated by high temperature, acidic pH and various proteases *in vitro* (Annes et al. 2003). However, the importance of these mechanisms *in vivo* remain to be determined. Recently, a fundamental role for members of the av integrin family in activating TGFb has been identified.

Integrins are a family of transmembrane receptors composed of an α and β subunit, with 24 different integrins expressed in mammals (Humphries et al. 2004). Integrins are important adhesion and signalling receptors, mediating both cell–cell and cell-extracellular matrix adhesion and conveying bi-directional signals across the plasma membrane (Hynes 2002). Several integrins are also capable of binding to a classical tri-amino acid binding motif, Arg-Gly-Asp (RGD) present in the LAP region of TGFβ1 and TGFβ3. *In vitro* binding studies have suggested that members of the integrin αv family (αvβ1, αvβ3, αvβ5, αvβ6, αvβ8) and integrin α8β1 can interact with the RGD site of LAP in latent TGFβ1 and 3 (Worthington et al. 2011a). Although there is no evidence that either αvβ1 or α8β1 binding to latent TGFβ can result in activation, strong data now exist suggesting a critical role for other αv integrins in regulating TGFβ activity and function during health and disease.

Seminal work by John Munger's group first proposed a fundamental role for integrins in activating TGFβ1 to control immune homeostasis. Knock-in mice were generated that expressed latent TGFβ1, but with a point mutation in the RGD integrin binding site to RGE. Thus, mice expressed latent TGFβ1 but in a form that could not be bound to or activated by integrins (TGFβ1^RGD→RGE^ mice) (Yang et al. 2007). Despite expressing similar levels of latent TGFβ1 to control animals, TGFβ1^RGD→RGE^ mice showed a remarkably similar phenotype to mice completely lacking TGFβ1 production, dying from multi-organ inflammation early in life (Yang et al. 2007). Subsequent work has shown that integrins αvβ6 and αvβ8 are the key activators of TGFβ1 in the steady state immune system, as a combined lack of function of these integrins recapitulates the phenotype seen in TGFβ1−/− and TGFβ1^RGD→RGE^ mice (Aluwihare et al. 2009). Thus, integrins αvβ6 and αvβ8 play a non-redundant role in the activation of TGFβ1 in the immune system which is required to prevent self-harmful immune responses.

## Integrin αvβ8-mediated TGFβ activation in the immune system: a crucial pathway in the regulation of immune homeostasis

After establishing a crucial role for integrins αvβ6 and αvβ8 in activating TGFβ *in vivo* to maintain immune homeostasis, an important question became where are these integrins expressed and how does activation of TGFβ regulate immunity to prevent inflammation? Expression of integrin αvβ6 is normally restricted to epithelial cells (Busk et al. 1992), suggesting an important role for epithelial cell-activated TGFβ in regulating immune cells to maintain homeostasis. Expression of integrin αvβ8 is more wide-spread, with expression seen in locations such as cells of the central nervous system, vascular epithelial cells and astrocytes in the brain (Milner et al. 1997; Nishimura et al. 1998; Zhu et al. 2002; Cambier et al. 2005), mesangial cells in the kidney (Khan et al. 2011) and fibroblasts and epithelial cells in the airway (Araya et al. 2006). Interestingly, integrin αvβ8 is expressed by cells of the immune system, most prominently in CD4+ T-cells and in DCs (Travis et al. 2007).

To reveal potential function of integrin-mediated TGFβ activation by cells of the immune system, conditional KO mice were generated by crossing mice expressing a conditional floxed β8 integrin allele (Proctor et al. 2005) with mice expressing Vav1-Cre to delete integrin αvβ8 in all leukocytes. As the β8 subunit only forms an integrin heterodimer with αv, this approach allowed specific deletion of integrin αvβ8. Such mice developed an age-related wasting disorder associated with T-cell activation and aberrant T-cell associated antibody production and by ∼6 months of age developed severe colitis (Travis et al. 2007). Similar results were observed in mice lacking all αv integrins in leukocytes (Lacy-Hulbert et al. 2007). Thus, expression of integrin αvβ8 by leukocytes is key to maintaining T-cell homeostasis and preventing inflammation of the intestine.

Additional work determined the cell type of the immune system that expressed functionally important integrin αvβ8. Lack of integrin αvβ8 expression in T-cells (using CD4-Cre-mediated deletion of a conditional β8 allele) caused no phenotype in mice (Travis et al. 2007). However, when integrin αvβ8 was deleted in CD11c+ cells, which are predominantly DCs (*via* expression of CD11c-Cre), mice developed an identical wasting and inflammatory disorder to mice lacking the integrin on all leukocytes (Travis et al. 2007). The phenotype was correlated with a decreased ability of DCs to activate TGFβ, and also a reduced level of Foxp3+ Tregs in the large intestine (Travis et al. 2007). Similar results were obtained when all αv integrins were deleted from myeloid cells (using LysM-Cre) (Lacy-Hulbert et al. 2007). Thus, integrin αvβ8-mediated TGFβ activation by CD11c+ DCs is a crucial pathway in the maintenance of immune homeostasis in the intestine.

### A key role for integrin αvβ8-mediated TGFβ activation by specialised DCs of the intestine

The intestine is a site of high immune load, given the trillions of microorganisms that form the microflora, and the diverse array of dietary antigens. Specialised immune pathways therefore exist in the intestine to promote tolerance in the face of the heavy antigenic load, with an important role for TGFβ (Maloy and Powrie 2011). Interestingly, a recently identified tolerogenic subset of DCs in the intestine, marked by expression of CD103, are specialised to induce Foxp3+ Tregs. This enhanced Treg induction is linked to their enhanced ability to produce retinoic acid, a metabolite of Vitamin A, and is TGFβ-dependent (Coombes et al. 2007; Sun et al. 2007). However, how TGFβ was activated in the intestine to drive Treg induction was not understood. We have recently shown that CD103+ intestinal DCs, as well as producing more retinoic acid, are specialised to activate TGFβ which correlates with high expression of integrin αvβ8 (Worthington et al. 2011b). Indeed, enhanced TGFβ activation by CD103+ gut DCs promotes Foxp3+ Treg induction even when the actions of retinoic acid are blocked, and loss of integrin αvβ8 expression by CD103+ intestinal DCs ablates their enhanced ability to activate TGFβ and induce Foxp3+ Tregs (Worthington et al. 2011b; Païdassi et al. 2011). Thus, integrin αvβ8-mediated TGFβ activation is upregulated by specialised DC subsets in the intestine, and this pathway is crucial in maintaining immune homeostasis in the gut.

Interestingly, although mice lacking the TGFβ-activating integrin αvβ8 on immune cells develop autoimmunity, this is not as severe as mice that lack global function of integrins αvβ6 and αvβ8 (Aluwihare et al. 2009). Global integrin αvβ6−/− mice develop only mild inflammation of the lung and skin (Huang et al. 1996). Crossing these mice with mice lacking integrin αvβ8 on leukocytes, although speeding the onset of colitis compared to mice lacking integrin αvβ8 on leukocytes, does not result in rapid multi-organ inflammation observed in the total absence of integrins αvβ6 and αvβ8 (Dean Sheppard and Mark Travis, unpublished data). Thus, expression of integrin αvβ8 on non-leukocyte cells appears to be important in the maintenance of immune homeostasis. Data does suggest that integrin αvβ8 can activate TGFβ in a number of non-immune cell types including airway fibroblasts and epithelial cells (Mu et al. 2002; Araya et al. 2006, 2007; Kitamura et al. 2011), astrocytes (Cambier et al. 2005; Hirota et al. 2011), and Muller glial cells and neurons (Arnold et al. 2012). However, the exact non-immune cell types required for integrin αvβ8-mediated TGFβ activation to maintain immune homeostasis remain to be determined.

## Integrin αvβ8-mediated TGFβ activation by dendritic cells in inflammatory disorders

In addition to playing an important role in dampening immune responses to maintain homeostasis, activation of TGFβ by DCs is crucial in promoting inflammation during autoimmunity. As mentioned earlier, as well as having important suppressive effects on CD4+ T-cells, TGFβ can also promote inflammatory T-cell responses by promoting differentiation of Th17 cells. It is now apparent that integrin αvβ8-mediated TGFβ activation by DCs is important in the development of Th17 cells. DCs that lack integrin αvβ8 expression have a reduced ability to induce Th17 cells *in vitro*, and this defect can be rescued by the addition of active TGFβ (Melton et al. 2010). Mice lacking integrin αvβ8 expression on DCs also have reduced numbers of Th17 cells *in vivo*, and are protected from symptoms during EAE, a model of autoimmunity known to involve Th17 cells (Melton et al. 2010). Similar results are observed in mice lacking all αv integrins on myeloid cells, and when integrin-latent TGFβ interactions were blocked by administration of an RGD peptide (Acharya et al. 2010). Thus, TGFβ activation by DCs can drive Th17-mediated inflammation during autoimmune disease.

TGFβ activation by DCs is also important in the development of allergic disease in the lung. Mice lacking integrin αvβ8 on DCs display reduced airway hyper-responsiveness (AHR), a hallmark of allergic asthma, during lung antigen challenge models (Kudo et al. 2012). Reduced AHR is associated with impaired Th17 cell induction, which results in attenuated IL-17-mediated smooth muscle contraction in the airways (Kudo et al. 2012). Interestingly, lung inflammation observed during challenge was similar between control mice and mice lacking integrin αvβ8 on DCs. Thus, Th17 cells induced in the lung *via* DC-mediated TGFβ activation appear to play a specific function in regulating AHR during pulmonary antigen challenge rather than driving a broader inflammatory response.

In addition to driving activation of TGFβ *via* integrin αvβ8, it has recently been shown that DCs themselves can be influenced by αvβ8-mediated TGFβ activation in the lung. Thus, during airway remodelling (a hallmark of lung diseases such as asthma and chronic obstructive pulmonary disease) airway fibroblasts upregulate integrin αvβ8 to activate TGFβ (Kitamura et al. 2011). This increased activation of TGFβ promotes production of the chemokines CCL2 and CCL20 by the fibroblasts, enhancing DC migration to the lung to boost adaptive immune responses (Kitamura et al. 2011). Given the known importance of integrin αvβ6 expression by lung epithelial cells in regulation of lung pathology during various pulmonary diseases (Sheppard 2004), and recent data suggesting an important role for integrin αvβ5-mediated TGFβ activation by airway smooth muscle cells in mediating pathology during asthma (Tatler et al. 2011), it appears that there are multiple integrin-mediated mechanisms to regulate TGFβ activity in the lung during disease. Thus, it will be interesting to determine potential cross-talk between these related mechanisms of TGFβ activation in the lung, and how this impacts on lung pathology during pulmonary disease.

## Conclusions

**Fig. 2:**
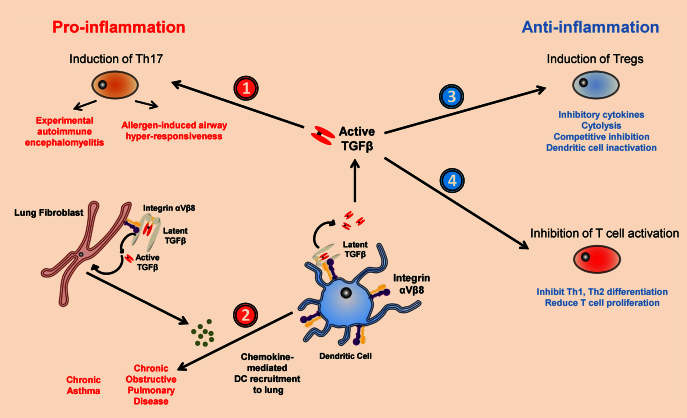
Integrin αvβ8-mediated TGFβ activation is mediated by, and acts upon DCs to regulate pro- and anti-inflammatory immune responses. Latent TGFβ binds integrin αvβ8 integrin *via* an RGD motif in the LAP region of the latent complex, resulting in the release of active TGFβ which can orchestrate pro- or anti-inflammatory responses. Pro-inflammatory immunity is stimulated when (1) Th17 cells are induced from naive CD4+ T-cells DC-mediated TGFβ activation *via* integrin αvβ8, which can result in diseases such as EAE and AHR and (2) TGFβ is activated by lung fibroblast-expressed integrin αvβ8, with TGFβ acting in an autocrine manner to stimulate chemokine secretion and DC recruitment to the lung. Anti-inflammatory immunity is stimulated when TGFβ activated by DC-expressed integrin αvβ8: (3) induces Foxp3+ Tregs and (4) directly inhibits T-cell activation.

Given its crucial and multi-functional role in controlling immunity, it is unsurprising that the action of TGFβ is carefully regulated, not only at the level of cytokine production but also by activation of the latent complex. Recent data highlighting a crucial role for integrins in activating TGFβ has highlighted not only a novel function for integrin family members *in vivo*, but also a key immunoregulatory mechanism that controls both tolerogenic and inflammatory immune responses. The finding that DCs are especially important in activating TGFβ *via* integrin αvβ8 has uncovered a novel pathway that allows intricate regulation of T-cell responses during health and disease (Fig. 2). However, many important questions remain. How does integrin binding to the latent complex of TGFβ result in TGFβ activation? Mechanisms proposed include contraction induced stretching of the latent complex after integrin engagement (for integrins αvβ3, αvβ5 and αvβ6) and protease recruitment and cleavage of the complex (for integrin αvβ8) (Worthington et al. 2011a). However, whether these mechanisms are important *in vivo*, and whether they are conserved between different cell types in different tissues of the body remains to be seen. Additionally, are there subsets of cells throughout the body that are specialised to activate TGFβ *via* different integrin heterodimers, akin to the upregulation of integrin αvβ8 seen in CD103+ DCs in the intestine? Furthermore, how are these pathways altered during disease? Regardless, TGFβ-activating integrins are becoming potential therapeutic targets in a range of immunological disorders, to either stimulate anti-inflammatory pathways or inhibit pro-inflammatory pathways during progression of chronic disease.
